# Effects of Platycodin D on Proliferation, Apoptosis and PI3K/Akt Signal Pathway of Human Glioma U251 Cells

**DOI:** 10.3390/molecules191221411

**Published:** 2014-12-19

**Authors:** Chong Xu, Guibo Sun, Guangxin Yuan, Rui Wang, Xiaobo Sun

**Affiliations:** 1 Key Laboratory of Bioactive Substances and Resources Utilization of Chinese Herbal Medicine, Ministry of Education, Institute of Medicinal Plant Development, Chinese Academy of Medical Sciences and Peking Union Medical College, Beijing 100193, China; 2 Pharmaceutical College, Beihua University, Jilin 132013, China; 3 School of Pharmaceutical Sciences, Jilin University, Changchun 130021, China

**Keywords:** platycodin D, glioma U251 cells, proliferation, apoptosis, PI3K/Akt

## Abstract

Effects of platycodin D (PD) on the proliferation, apoptosis and PI3K/Akt signaling pathway of human glioma U251 cells were investigated. Glioma U251 cells were treated with PD at final concentrations of 0, 16.3, 40.8, 81.6, 163.2 μM, and inhibition rate, early and late apoptotic rate, apoptotic index, expression of apoptosis-related proteins and phosphorylation of the PI3K/Akt signaling pathway were evaluated. The results showed that compared with the control group, PD could increase the proliferation inhibition rate of U251 cells in a dose- and time -dependent manner; PD could also elevate the early and late apoptotic rate, apoptotic index and the level of pro-apoptotic proteins of glioma U251 cells, such as Bax and cleaved caspase-3, but lower the level of apoptosis inhibitory protein, such as Bcl-2; PD could increase the ratio of G_0_/G_1_ phase U251 cells, and lower the proportion of Sphase U251 cells and the ratio of G_2_/M phase U251 cells; PD could reduce the ratio of p-Akt/Akt. The results indicate that PD can inhibit the proliferation, induce the apoptosis and cause the cell cycle arrest in human glioma U251 cells, which may be related to the inhibition of PD on the activation of PI3K/Akt signaling pathway.

## 1. Introduction

Glioma is the most common intracranial malignant tumor developing in the neuroectoderm. Its incidence accounts for 40%–50% of all intracranial tumors, and it is characterized by high recurrence, high mortality and low cure rates [1]. Glioma includes astrocytoma, oligodendroglioma, ependymoma and choroid plexus papilloma, of which the incidence of astrocytoma developing from astrocytes or astrocyte precursors is highest [2,3]. Although significant progress in the treatment of gliomas with surgery, radiotherapy and chemotherapy has been made, the clinical treatment of gliomas is still far from satisfactory due to their multiple types, invasiveness and difficulty to completely remove them with surgery [4]. The treatment of gliomas with post-operative radiotherapy and chemotherapy is generally accepted, but the tolerance of tumor cells to the radiotherapy may promote the relapse of residual nidus [5]; Numerous anticancer drugs have been studied and developed at home and abroad, but they can induce more adverse reactions and their therapeutic effects are not satisfactory [6,7]. In recent years, many scholars have come to believe that the anti-tumor treatment possible with Traditional Chinese Medicine is unique, and the screening of anti-tumor active ingredients and lead compounds from Traditional Chinese Medicines has become a hot topic in this field [8,9]. Currently, anti-cancer agents derived from botanicals such as paclitaxel, camptothecin,* etc.*, which have become the drugs as the first choice in many tumor therapies, account for more than 30% of all anti-cancer drugs [10,11]. The abundance and variety of medicinal plants in China are rich, so it is more important to screen anti-tumor active ingredients from Traditional Chinese Medicines, confirm their anti-tumor effects and clarify their mechanisms for action for the research and development of new and better anticancer drugs.

**Figure 1:**
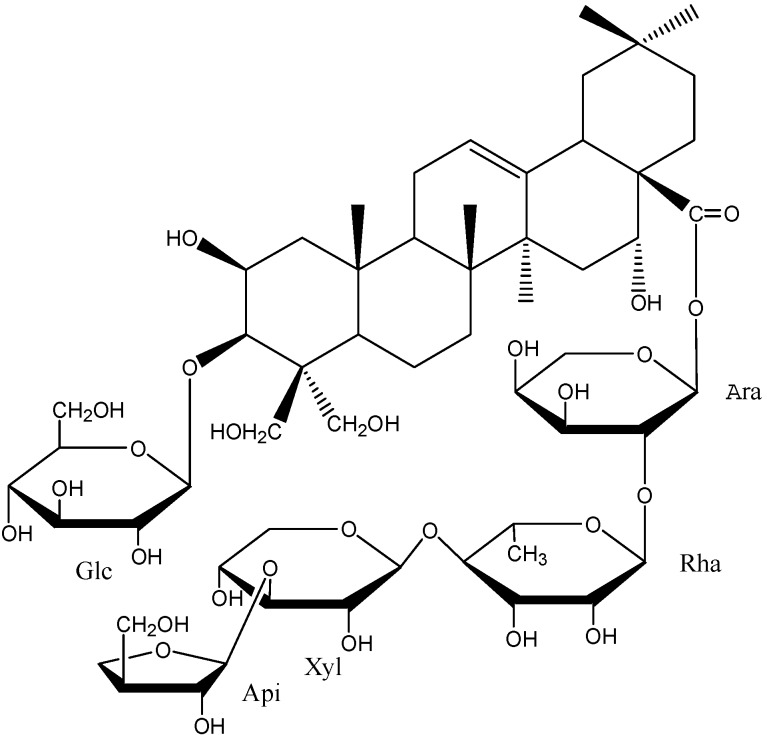
The chemical structure of platycodin D (C_57_H_92_O_28_, M.W. 1225).

*Platycodon grandiflorum*, a Chinese herbal medicine, is the dried root of a Campanulaceae plant. It is one of most important medicinal and edible plants in China and other Asian countries, widely used in food and for the treatment of bronchitis, asthma, tuberculosis and other chronic diseases [12,13]. Modern pharmaceutical research has demonstrated that triterpenoid saponins are the main active ingredients in this herbal medicine [14,15,16] and that platycodin D (PD, Figure 1) is the most important triterpenoid saponin-like ingredient extracted from *P. grandiflorum*. As the main effective ingredient in *P. grandiflorum*, PD has been shown to have some good anti-inflammation [17], anti-virus [18], heptoprotection [19], immunoregulation [20] and anti-tumor [21] pharmacological activities. There have been some reports on the inhibitory effects of platycodin D on liver cancer [22], ovarian cancer [23], breast cancer [24], gastric cancer [25], and leukemia [26], but none on the effect of PD on glioma and the related mechanisms through which PD can exert its tumor inhibition. In this study, the effect of PD on the proliferation and the apoptosis of human glioma U251 cells was investigated, and an attempt was made to study its mechanism of action by exploring the relationship between the effect of PD and cell cycle, and the apoptosis-related signaling pathway, in order to provide an experimental basis for the research and the application of PD in the treatment of glioma.

## 2. Results and Discussion

### 2.1. Effects of Different Concentrations of PD on the Proliferation of Human Glioma U251 Cells

**Figure 2:**
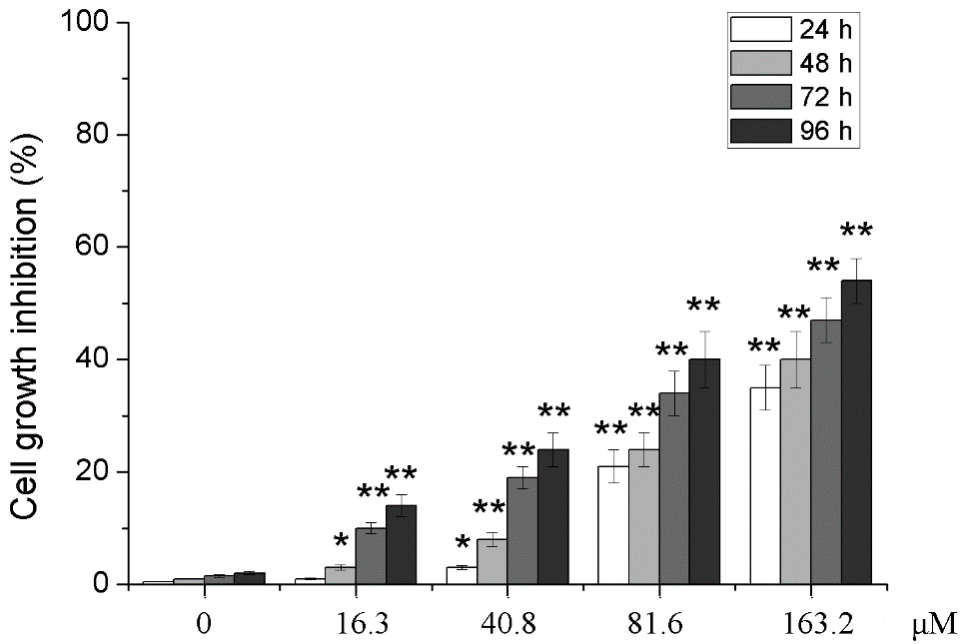
Effects of PD on cell growth inhibition of U251. U251 cells were treated with 0, 16.3, 40.8, 81.6 and 163.2 μM of PD for 24, 48, 72 and 96 h. Cell growth inhibition was measured by using MTT assay. Each value is presented as mean ± SD (*n* = 3). * *p* < 0.05 compared with 0 μM PD; ** *p* < 0.01 compared with 0 μM PD.

After U251 cells were treated with 0, 16.3, 40.8, 81.6 and 163.2 μM of PD, the MTT assay was used to observe the effect of different concentration of PD on the proliferation activity of U251 cells. As shown in Figure 2, in addition to 16.3 μM of PD at 24 h, inhibition rates of different concentrations of PD on the proliferation activity of U251 cells at 24, 48, 72 and 96 h after the treatment were significantly higher than those of 0 μM of PD at the same time points (*p* < 0.05 or *p* < 0.01), and the inhibition of PD presented an approximate dose- and time-dependent manner.

### 2.2. Effects of Different Concentrations of PD on the Apoptotic Rate of Human Glioma U251 Cells

**Figure 3:**
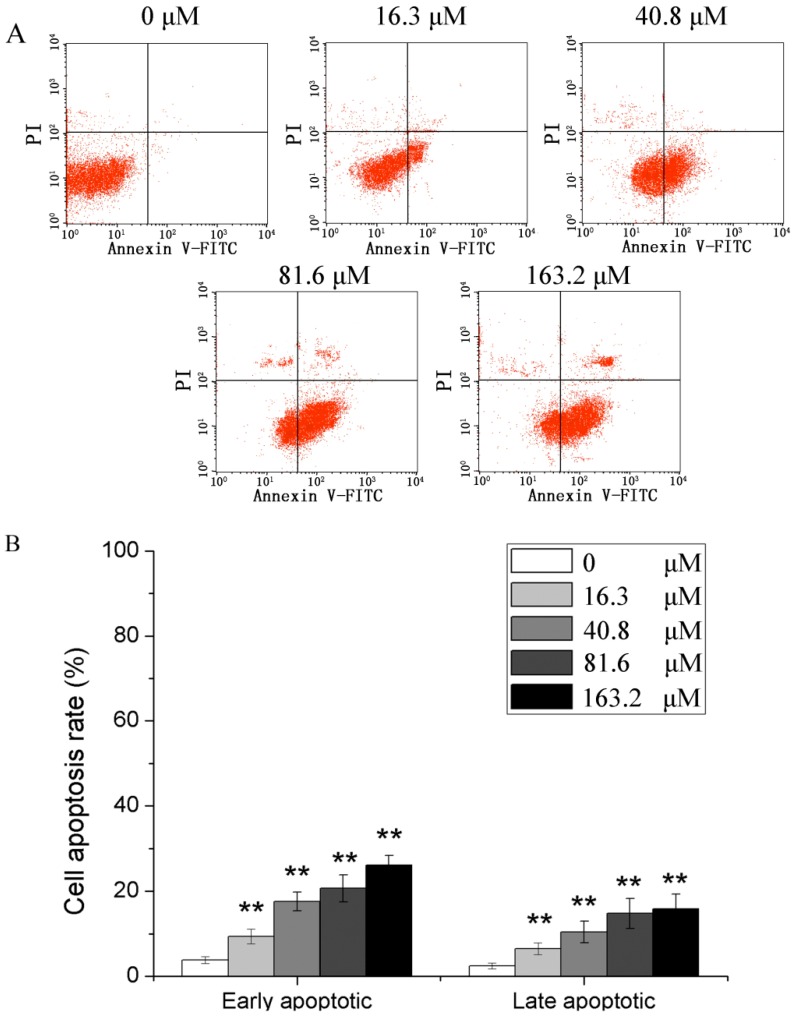
PD induced apoptosis in U251 cells. (**A**) flow cytometric analysis; (**B**) cell apoptosis rate. U251 cells were treated with 0, 16.3, 40.8, 81.6 and 163.2 μM of PD for 48 h. Then they were stained with FITC-conjugated Annexin V and PI for flow cytometric analysis. Each value is presented as mean ± SD (*n* = 3). ** *p* < 0.01 compared with 0 μM PD.

After U251 cells were treated with 0, 16.3, 40.8, 81.6 and 163.2 μM of PD for 48 h, Annexin V-FITC/PI double staining flow cytometry was applied to detect the apoptotic rates. The results (Figure 3) showed that PD could increase early and late apoptotic rates of U251 cells early, and the apoptotic rates of 0, 16.3, 40.8, 81.6 and 163.2 μM of PD were significantly higher than those of 0 μM of PD (*p* < 0.01).

### 2.3. Effects of Different Concentrations of PD on the Apoptotic Index Human Glioma U251 Cells

**Figure 4:**
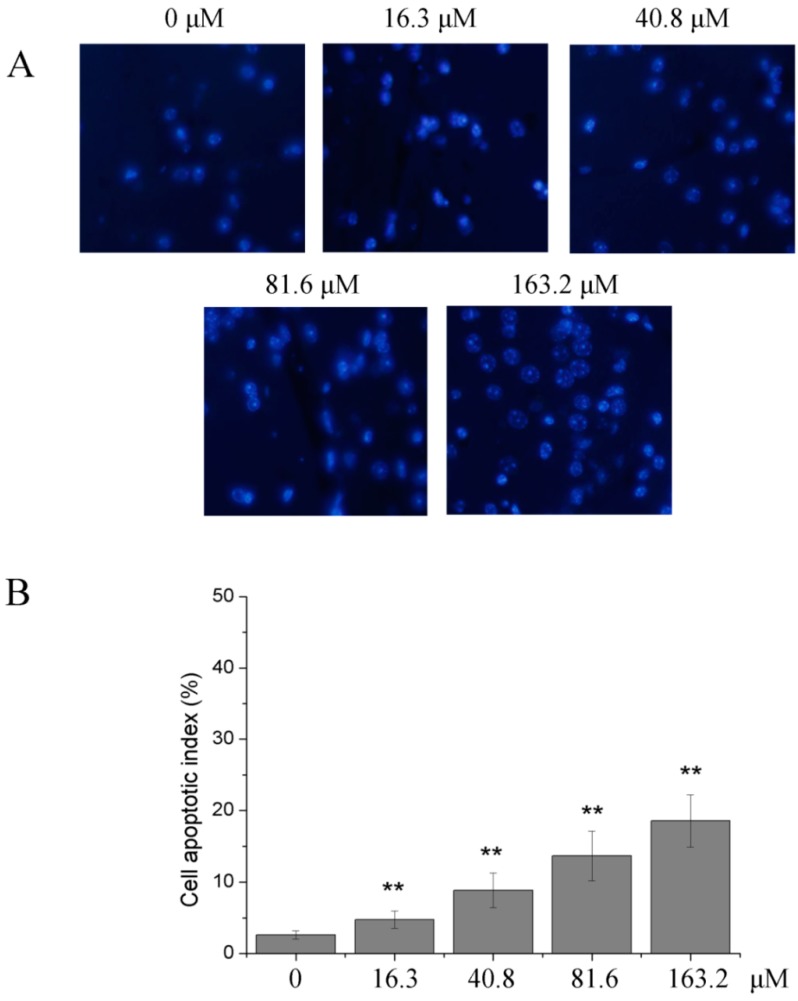
The effect of PD on cells apoptosis index in U251 cells. (**A**) Hoechst 33258 staining (×200); (**B**) cell apoptosis index. U251 cells were treated with 0, 16.3, 40.8, 81.6 and 163.2 μM of PD for 48 h. Then they were stained with Hoechst 33258. Each value is presented as mean ± SD (*n* = 3). ** *p* < 0.01 compared with 0 μM PD.

After U251 cells were treated with 0, 16.3, 40.8, 81.6 and 163.2 μM of PD for 48 h, Hoechst staining detection showed that the apoptotic indexes in the 16.3, 40.8, 81.6 and 163.2 μM PD groups which were in turn 2.12%, 6.24%, 11.03% and 15.91 (*p* < 0.01), were significantly higher than those in the 0 μM PD group, indicating that PD could increase the apoptotic index of U251 cells in a dose-dependent way. The data are shown in Figure 4.

### 2.4. Effects of Different Concentrations of PD on the Expression of Apoptosis-Related Genes in Human Glioma U251 Cells

**Figure 5:**
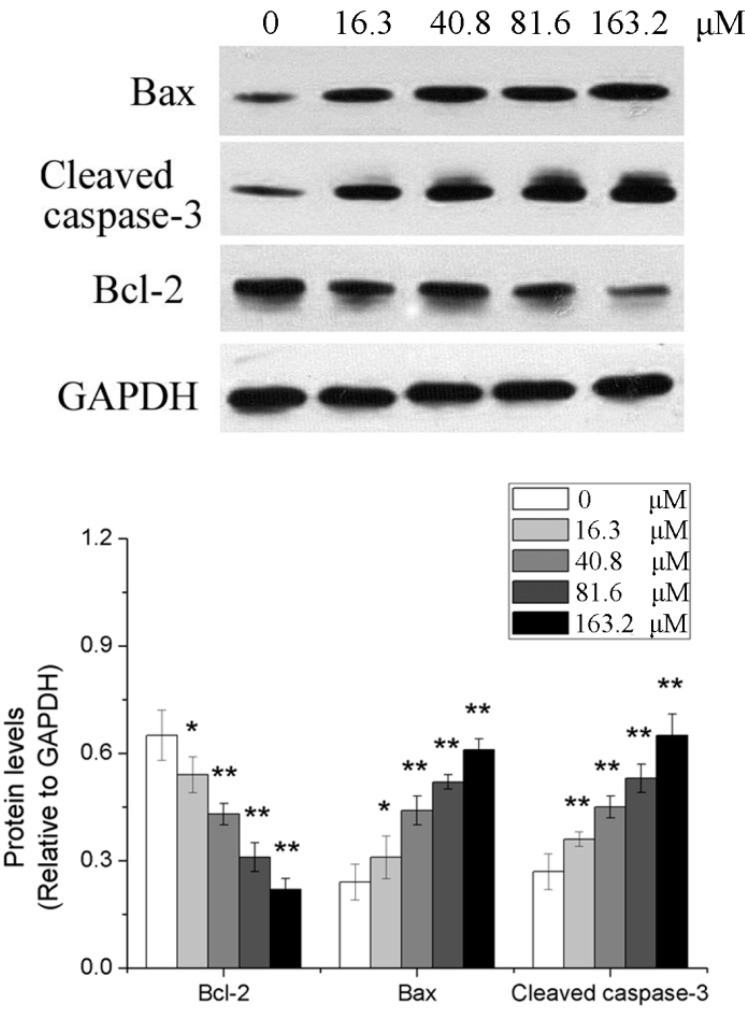
The effect of PD on the expressions of Bcl-2, Bax and cleaved caspase-3 in U251 cells. U251 cells were treated with 0, 16.3, 40.8, 81.6 and 163.2 μM of PD for 48 h. Then the expressions of target protein were evaluated by western blot assay. Each value is presented as mean ± SD (*n* = 3). * *p* < 0.05 compared with 0 μM PD; ** *p* < 0.01 compared with 0 μM PD.

After U251 cells were treated with 0, 16.3, 40.8, 81.6 and 163.2 μM of PD for 48 h, western blotting analysis showed that compared with those in the 0 μM group, Bax and cleaved caspase-3 protein levels were elevated, but Bcl-2 protein levels were reduced in the other PD groups (*p* < 0.05 or *p* < 0.01). The results are shown in Figure 5.

### 2.5. Effects of Different Concentrations of PD on the Cell Cycle of Human Glioma U251 Cells

**Figure 6:**
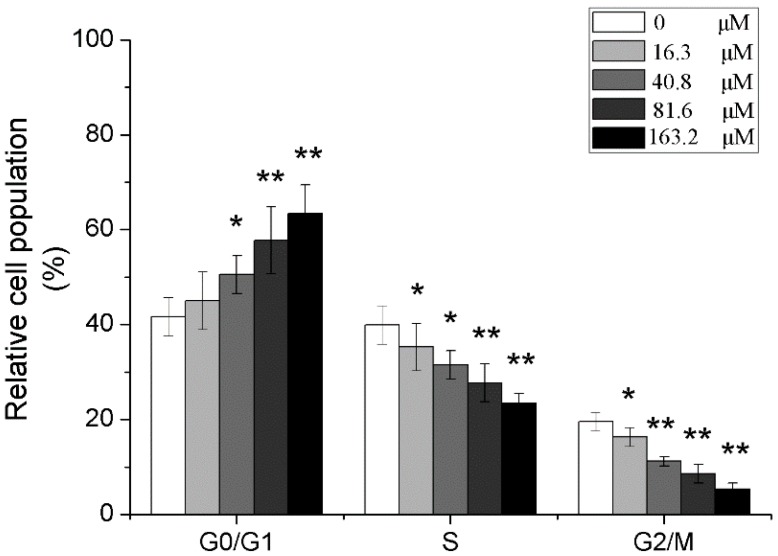
The effect of PD on the cell cycle in U251 cells. U251 cells were treated with 0, 16.3, 40.8, 81.6 and 163.2 μM of PD for 48 h. Then they were stained with PI for flow cytometric analysis. Each value is presented as mean ± SD (*n* = 3). * *p* < 0.05 compared with 0 μM PD; ** *p* < 0.01 compared with 0 μM PD.

After U251 cells were treated with 0, 16.3, 40.8, 81.6 and 163.2 μM of PD for 48 h, the detection with PI staining flow cytometry found that ratios of G_0_/G_1_ phase cells were increased in all PD dose groups except for those in the 0 μM PD group, in which those in 40.8, 81.6 and 163.2 μM groups were significantly increased (*p* < 0.05 or *p* < 0.01); meanwhile, PD could also significantly reduce the ratio of S and G_2_/M phase cells, which was dose-dependent (*p* < 0.05 or *p* < 0.01), as shown in Figure 6.

### 2.6. Effects of Different Concentrations of PD on the PI3K/Akt Signaling Pathway of Human Glioma U251 Cells

**Figure 7:**
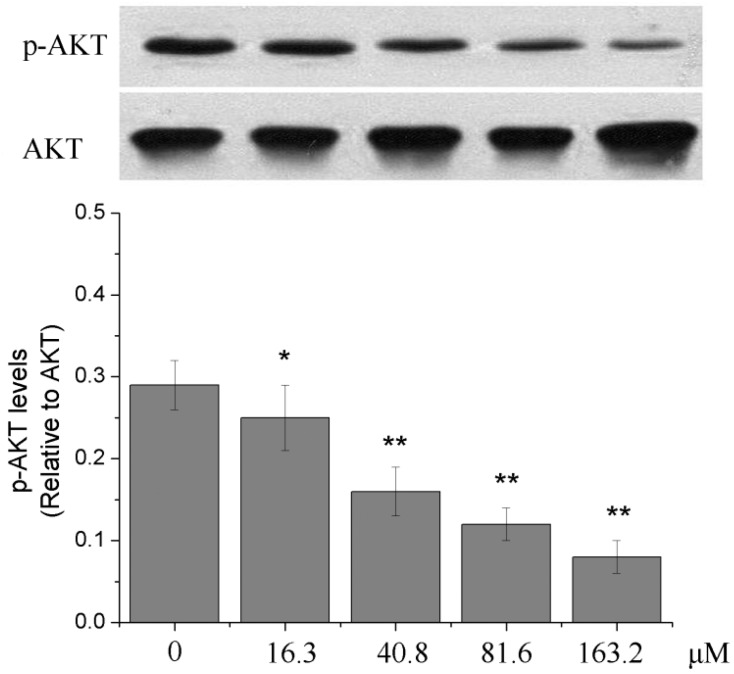
The effect of PD on the PI3K/Akt signaling pathway in U251 cells. U251 cells were treated with 0, 16.3, 40.8, 81.6 and 163.2 μM of PD for 48 h. Then the expressions of p-AKT and AKT were evaluated by western blot assay. Each value is presented as mean ± SD (*n* = 3). * *p* < 0.05 compared with 0 μM PD; ** *p* < 0.01 compared with 0 μM PD.

After U251 cells were treated with 0, 16.3, 40.8, 81.6 and 163.2 μM of PD for 48 h, the ratio of p-AKT/AKT evidently was reduced with the increase of concentrations of PD (*p* < 0.05 or *p* < 0.01). The results indicated that PD could inhibit the activation of PI3K/Akt signal pathway, as shown in Figure 7.

### 2.7. Discussion

Currently, research on the anti-tumor activity of the active ingredients from *P. grandiflorum* has become a hot spot. PD is a triterpenoid extracted from the Traditional Chinese Medicine *P. grandiflorum* [27]. Many studies have verified that PD has a broad cytotoxic activity and effectively inhibits the growth of a variety of tumor cells, such as liver cancer, ovarian cancer, breast cancer, stomach cancer and leukemia and other cancers [22,23,24,25,26]. At present, there has been no study yet to show the biological activity of PD in human glioma U251 cells, so in this study, human glioma U251 cells were treated with PD for the observation on its activity against human glioma. The study found that PD could inhibit the proliferation of U251 cells, in which the inhibition was dose- and time- dependent at dose range from 40.8–163.2 μM, the proliferation inhibition rates of these dosages of PD were higher than those at 0 μM of PD. These results indicate that PD can inhibit the proliferation of human glioma U251 cells.

Inducing the apoptosis of tumor cells is an important way through which anti-tumor drugs exert their therapeutic effects, and the anti-tumor effect of plant extracts and the relationship between their effects and the induction of tumor cell apoptosis is currently a research hot spot [28,29,30]. Apoptosis is a cell suicide phenomenon regulated by genes and biologically significant in the occurrence and development of tumors, and many chemotherapeutic drugs exert their inhibitory effects on tumors by inducing the apoptosis of tumor cells [31,32]. In our study, an Annexin V-FITC/PI double staining flow cytometry and a Hoechst staining were applied to investigate effects of PD on the apoptosis of human glioma U251 cells. The results showed that compared with the none-PD treatment of U251 cells, the treatment of U251 cells with PD could significantly increase the apoptosis and apoptotic index of U251 cells, indicating that PD can induce the apoptosis of U251 cells.

Two pathways including mitochondria and death receptors are primarily involved in apoptosis, and the mitochondrial pathway is composed of Bid and Bad that are members of Bcl-2 family containing the BH3 domain and can be activated after they receive intracellular death signals [33]. The Bcl-2 family proteins are important regulators of mitochondrial apoptosis, including two types of proteins with opposite functions, namely, inhibition or promotion. The interaction between Bcl-2 family protein members plays a regulatory role in the mitochondrial-mediated apoptosis [34,35]. The interaction between anti-apoptotic binding protein Bcl-2 and pro-apoptotic protein Bax or Bak that binds to the outer membrane surface of mitochondria or exists in the cytoplasm can activate the caspase pathway, ultimately inducing apoptosis [36]. The results of this study showed that PD could reduce the Bcl-2 protein level of human glioma U251 cells, and elevate the level of Bax and cleaved caspase-3 protein, indicating that PD may induce the apoptosis of U251 cells by changing the expression of Bcl-2 and Bax proteins to activate the caspase pathway.

The cell cycle of normal cells is a strictly regulated process and cell cycle disorder is a basic characteristic of tumor cells so that anti-tumor effects can be exerted by disturbing the cell cycle of tumor cells [37,38,39]. This study found that PD could affect the cell cycle of U251 cells, in which the ratio of U251 cells treated with different concentrations of PD for 48 h was lower in S phase and G_2_/M phase, while that in G_0_/G_1_ phase was higher, suggesting that PD can arrest the cells in the G_0_/G_1_ phase. Besides the excessive proliferation of cells and the cell cycle abnormality, the occurrence of malignancies is also related to the reduction of cell apoptosis [40]. The phosphatidyl-inositol 3-kinase/serine-threonine kinase (PI3K/Akt) signaling pathway plays an important role in the regulation of cell biological behaviors, such as the proliferation and the apoptosis, in which this pathway is in an active state in most tumors [41,42]. It is reported that many chemotherapeutic drugs can exert their anti-tumors effects by blocking this pathway [43,44]. Akt is an important component of PI3K/Akt signaling pathway; when this pathway is activated, the activity of PI3K is enhanced, the phosphorylated form of Akt is predominant and the ratio of p-Akt (p-Akt/Akt) is increased [45]. The experimental results showed that compared with the treatment without PD, the treatment of U251 cells with PD could reduce the ratio of p-Akt/Akt, suggesting that PD may inhibit the activation of PI3K/Akt signaling pathway of U251 cells.

## 3. Experimental Section

### 3.1. Materials and Chemicals

PD (purity > 98%) was purchased from Best-Reagent (Chengdu, China) and human glioma cell line (U251) was purchased from ATCC (Rockefeller, MA, USA). Fetal calf serum, trypsin and RPMI 1640 were obtained from Gibco Co. (Grand Island, NY, USA) and MTT, PI and Triton X-100 were obtained from Sigma Chemical Co. (St. Louis, MO, USA). Antibodies of Bax, Bcl-2, Cleaved caspase-3, AKT, and p-AKT were gained from Cell Signaling Technology (Danvers, MA, USA). All of other reagents were of analytical grade from Sinopharm Chemical Reagent Co (Beijing, China).

### 3.2. Detection of Cell Proliferation Activity

Thawed U251 cells were routinely cultured under the condition of 37 °C and 5% CO_2_ in a culture medium containing 15% fetal bovine serum, the medium was changed every other 24 h, a light microscope was used to observe the cells, and the cells were subcultured 3–5 generations with 0.25% trypsin digestion and subculture when the confluence reached 80%–90%. Logarithmic growth phase U251 cells were seeded in a 96-well culture plate at a density of 1 × 10^5^/well; 24 h later, PD at its final concentration of 0, 16.3, 40.8, 81.6 and 163.2 μM was in turn added to the culture medium respectively, in which the well containing 0 μM of PD was taken as the control group and the remaining wells containing different concentrations of PD were the experiment groups. All the cells were cultured under normal conditions, 10 μL of 5 mg/mL MTT solution were added to each well at 48, 72 and 96 h after the culture; 4 h later, absorbance values (A) of each group were recorded with a microplate reader (Thermo, Franklin, MA, USA) at the wavelength of 492 nm, which was repeated 3 times at the same time points for each concentration group and the proliferation inhibition rates were calculated according to the following formula:

Proliferation inhibition rate (%) = (1 − A_experiment_/A_control_) × 100%

### 3.3. Detection of Cell Apoptosis and Cell Cycle

The cell apoptosis was detected with an Annexin V-FITC/PI double staining flow cytometry and the cell cycle was observed with a PI single staining flow cytometry. U251 cells were seeded in 6-well culture plates at a density of 1 × 10^6^/well, and 24 h later, PD at the final concentration of 0, 16.3, 40.8, 81.6 and 163.2 μM was respectively added to the plates in turn, and the cells were cultured under conventional conditions; 24 h later, the cells were harvested and washed with PBS, Annexin V/FITC and PI were respectively added to them for the staining for 15 min, and then the cell apoptosis and cell cycle were detected with a flow cytometry (BD Biosciences, San Jose, CA, USA) and PI single staining flow cytometry separately, based on the kit instructions.

### 3.4. Detection of Apoptotic Index

U251 cells in a single cell suspension were seeded on sterile small slides placed at the bottom of plates in advance and were observed under a light microscope; after the cells adhered to the wall of plates, PD was added to them, in which the final concentrations of PD in wells were 0, 16.3, 40.8, 81.6 and 163.2 μM respectively; after the cells were cultured for 48 h, the slides were fixed with paraformaldehyde, then after washing with PBS, stained with Hoechst 33258 (5 μg/mL) staining at room temperature in the dark; 30 min later, they were observed under a fluorescence microscope (Nikon, Chiyoda-ku, Tokyo, Japan) and five high-power visual fields were randomly selected for each slide. The apoptotic index was calculated according to the following formula:

Apoptotic index = Number of apoptotic cells/total cells × 100%

### 3.5. Western Blotting Analysis

U251 cells treated with 0, 16.3, 40.8, 81.6 and 163.2 μM of PD for 48 h were centrifuged at 1000 r/min for 5 min. The cell lysate was added to the precipitated cells and the cells were lysed on an ice bath, and then the protein concentration was measured by BCA method; a quantitative protein was taken from each sample, and the transfer film was performed routinely with 10% SDS-PAGE gel electrophoresis and semi-transfer method (Bio-Rad, Hercules, CA, USA) after the protein was mixed with the loading buffer; according to the size of film, an appropriate amount of the primary antibody was added to it at a dilution ratio of 1:2000 in addition to the ratio of 1:1000 for Bax, which was incubated at 4 °C overnight; after the film was washed with TBST, the secondary antibody (1:3000) was added to, which was kept at room temperature for 2 h and then the coloration with ECL was conducted; Gel-Pro analyzer software was used to the optical density of each band, in which in addition to the p-Akt-related expression amount expressed in the ratio of Akt optical density, the rest are expressed in the ratio of optical density of the target protein to that of GAPDH.

### 3.6. Statistical Analysis

All results were expressed as mean ± SD. Data were analyzed by standard *t*-test. *p* values less than 0.05 or 0.01 were considered statistically significant.

## 4. Conclusions

PD can inhibit the proliferation, induce apoptosis and cause cell cycle arrest in human glioma U251 cells, which may be related to the inhibition of PD on the activation of PI3K/Akt signaling pathway.

## References

[1] Jovčevska I., Kočevar N. (2013). Glioma and glioblastoma-how much do we (not) know?. Mol. Clin. Oncol..

[2] Gurney J.G., Kadan-Lottick N. (2001). Brain and other central nervous system tumors: Rates, trends, and epidemiology. Curr. Opin. Oncol..

[3] Wrensch M., Minn Y. (2002). Epidemiology of primary brain tumors: Current concepts and review of the literature. Neuro Oncol..

[4] Partap S., Fisher P.G. (2007). Update on new treatments and developments in childhood brain tumors. Curr. Opin. Pediatr..

[5] Yaneva M.P., Semerdjieva M.L. (2010). Postoperative chemo-radiotherapy with temodal in patients with glioblastoma multiforme-survival rates and prognostic factors. Folia Med. (Plovdiv).

[6] Fernández A., Sessel S. (2009). Selective antagonism of anticancer drugs for side-effect removal. Trends Pharmacol. Sci..

[7] Perfetti V., Palladini G. (2007). Bortezomib-induced paralytic ileus is a potential gastrointestinal side effect of this first-in-class anticancer proteasome inhibitor. Eur. J. Gastroenterol. Hepatol..

[8] Park E.H., Kim Y.J. (2014). Stereospecific anticancer effects of ginsenoside Rg3 epimers isolated from heat-processed American ginseng on human gastric cancer cell. J. Ginseng Res..

[9] Auyeung K.K., Cho C.H. (2009). A novel anticancer effect of *Astragalus saponins*: Transcriptional activation of NSAID-activated gene. Int. J. Cancer.

[10] Ando M., Yonemori K. (2012). Phase I and pharmacokinetic study of nab-paclitaxel, nanoparticle albumin-bound paclitaxel, administered weekly to Japanese patients with solid tumors and metastatic breast cancer. Cancer Chemother. Pharmacol..

[11] Minelli R., Cavalli R. (2012). Nanosponge-encapsulated camptothecin exerts anti-tumor activity in human prostate cancer cells. Eur. J. Pharm. Sci..

[12] Nyakudya E., Jeong J.H. (2014). Platycosides from the Roots of *Platycodon grandiflorum* and Their Health Benefits. Prev. Nutr. Food Sci..

[13] Park S.J., Lee H.A. (2012). *Platycodon grandiflorus* alleviates DNCB-induced atopy-like dermatitis in NC/Nga mice. Indian J. Pharmacol..

[14] Fu W.W., Fu J.N. (2011). Platycoside O, a new triterpenoid saponin from the roots of *Platycodon grandiflorum*. Molecules.

[15] Li W., Zhang W. (2010). Platycoside N: A new oleanane-type triterpenoid saponin from the roots of *Platycodon grandiflorum*. Molecules.

[16] Choi Y.H., Yoo D.S. (2008). Platyconic acid A, a genuine triterpenoid saponin from the roots of *Platycodon grandiflorum*. Molecules.

[17] Jang K.J., Kim H.K. (2013). Anti-inflammatory effects of saponins derived from the roots of *Platycodon grandiflorus* in lipopolysaccharide-stimulated BV2 microglial cells. Int. J. Mol. Med..

[18] Kim J.W., Park S.J. (2013). Triterpenoid Saponins Isolated from *Platycodon grandiflorum* Inhibit Hepatitis C Virus Replication. Evid. Based Complement. Altern. Med..

[19] Lim J.H., Kim T.W. (2013). Protective effect of the roots extract of *Platycodon grandiflorum* on bile duct ligation-induced hepatic fibrosis in rats. Hum. Exp. Toxicol..

[20] Xie Y., Pan H. (2008). A promising balanced Th1 and Th2 directing immunological adjuvant, saponins from the root of *Platycodon grandiflorum*. Vaccine.

[21] Lee J.H., Oh E.K. (2013). Crude saponins from *Platycodon grandiflorum* induce apoptotic cell death in RC-58T/h/SA#4 prostate cancer cells through the activation of caspase cascades and apoptosis-inducing factor. Oncol. Rep..

[22] Qin H., Du X. (2014). Platycodin D, a triterpenoid saponin from *Platycodon grandiflorum*, induces G_2_/M arrest and apoptosis in human hepatoma HepG2 cells by modulating the PI3K/Akt pathway. Tumour Biol..

[23] Hu Q., Pan R. (2010). *Platycodon grandiflorum* induces apoptosis in SKOV3 human ovarian cancer cells through mitochondrial-dependent pathway. Am. J. Chin. Med..

[24] Yu J.S., Kim A.K. (2010). Platycodin D induces apoptosis in MCF-7 human breast cancer cells. J. Med. Food.

[25] Chun J., Joo E.J. (2013). Platycodin D induces anoikis and caspase-mediated apoptosis via p38 MAPK in AGS human gastric cancer cells. J. Cell. Biochem..

[26] Kim M.O., Moon D.O. (2008). Platycodin D induces apoptosis and decreases telomerase activity in human leukemia cells. Cancer Lett..

[27] Tada A., Kaneiwa Y. (1975). Studies on the saponins of the root of *Platycodon grandiflorum* A. De Candolle. I. Isolation and the structure of platycodin-D. Chem. Pharm. Bull. (Tokyo).

[28] Feng X., Li L. (2014). Dihydroartemisinin potentiates the anticancer effect of cisplatin via mTOR inhibition in cisplatin-resistant ovarian cancer cells: Involvement of apoptosis and autophagy. Biochem. Biophys. Res. Commun..

[29] Zar P.P., Yano S. (2014). *In vitro* anticancer activity of loquat tea by inducing apoptosis in human leukemia cells. Biosci. Biotechnol. Biochem..

[30] Lim J.H., Lee Y.M. (2014). Anticancer activity of hispidin via reactive oxygen species-mediated apoptosis in colon cancer cells. Anticancer Res..

[31] Wimardhani Y.S., Suniarti D.F. (2014). Chitosan exerts anticancer activity through induction of apoptosis and cell cycle arrest in oral cancer cells. J. Oral Sci..

[32] Shao J., Ma Z.Y. (2014). Thiosemicarbazone Cu (II) and Zn (II) complexes as potential anticancer agents: Syntheses, crystal structure, DNA cleavage, cytotoxicity and apoptosis induction activity. J. Inorg. Biochem..

[33] Estaquier J., Vallette F. (2012). The mitochondrial pathways of apoptosis. Adv. Exp. Med. Biol..

[34] Su J., Zhou L. (2014). Bcl-2 Family Proteins Are Involved in the Signal Crosstalk between Endoplasmic Reticulum Stress and Mitochondrial Dysfunction in Tumor Chemotherapy Resistance. Biomed. Res. Int..

[35] Renault T.T., Teijido O. (2013). Regulation of Bax mitochondrial localization by Bcl-2 and Bcl-x (L): Keep your friends close but your enemies closer. Int. J. Biochem. Cell Biol..

[36] Cory S., Adams J.M. (2002). The Bcl-2 family: Regulators of the cellular life or death switch. Nat. Rev. Cancer.

[37] Penthala N.R., Bommagani S. (2014). Heck products of parthenolide and melampomagnolide-B as anticancer modulators that modify cell cycle progression. Eur. J. Med. Chem..

[38] Neumann J., Boerries M. (2014). The natural anticancer compound rocaglamide selectively inhibits the G_1_-S-phase transition in cancer cells through the ATM/ATR-mediated Chk1/2 cell cycle checkpoints. Int. J. Cancer.

[39] Pandeti S., Sharma K. (2014). Synthesis of novel anticancer iridoid derivatives and their cell cycle arrest and caspase dependent apoptosis. Phytomedicine.

[40] Rosati A., Ammirante M. (2007). Apoptosis inhibition in cancer cells: A novel molecular pathway that involves BAG3 protein. Int. J. Biochem. Cell Biol..

[41] Su B., Gao L. (2014). A genome-wide RNAi screen identifies FOXO4 as a metastasis-suppressor through counteracting PI3K/AKT signal pathway in prostate cancer. PLoS One.

[42] Wang H., Duan L. (2014). Activation of the PI3K/Akt/mTOR/p70S6K pathway is involved in S100A4-induced viability and migration in colorectal cancer cells. Int. J. Med. Sci..

[43] Ryu Y.L., Jung K.H. (2014). Anticancer activity of HS-527, a novel inhibitor targeting PI3-kinase in human pancreatic cancer cells. Cancer Lett..

[44] Zhang X., Li X.R. (2013). Current status and future perspectives of PI3K and mTOR inhibitor as anticancer drugs in breast cancer. Curr. Cancer Drug Targets.

[45] Bhutani J., Sheikh A. (2013). Akt inhibitors: Mechanism of action and implications for anticancer therapeutics. Infect. Agents Cancer.

